# Supplementary material for: Risk factors for cardiovascular disease among Saudi students: Association with BMI, current smoking, level of physical activity, and dietary habits
Source: PLoS One. 2025 May 8;20(5):e0321206. doi: 10.1371/journal.pone.0321206 (PMC12061183; doi:10.1371/journal.pone.0321206)
Supplement: Attachment — Submitted filename: Response point-by-point.pdf [file pone.0321206.s002.pdf]

Response point-by-point

Reviewer1:

Thanks for the invitation to peer-review this manuscript. It was well written, and the topic was really important. I think this manuscript could be published in the current format.

Response:

I am extremely grateful for your kind words and thoughtful criticism. We are delighted to hear that you found the manuscript well written and the subject interesting. We are deeply grateful for your support and favorable assessment of our work. We look forward to disseminating our results to a wider audience, thanks in part to your help.

Reviewer 2:

Thank you for extending the invitation to review a manuscript for PLOS ONE. The manuscript addresses an important topic: the factors associated with cardiovascular diseases among students. However, there are several important issues within the manuscript that make it unsuitable for publication.

Comment 1:

The most important issue of this manuscript is that the low response rate to the invitations sent to the students 56.62%. The authors should provide their calculation regarding the minimum sample size needed in this study and then evaluate whether this high rate of non-response has affected their power of study or not. Besides, there might be non-response bias, meaning that those who didn't respond could differ systematically from those who did. This could affect the generalizability of the findings.

Response:

Thank you for this significant observation concerning response rate and the possibility of non-response bias. Please, note that the response rate of our study, 56.62%, exceeds the average response rate for online surveys, which is 44.1% (Wu et al., 2022).

In addition, and to guarantee the robustness of our findings, we conducted a power analysis before initiating the investigation, establishing a minimum sample size of 384 participants based on a 95% confidence level and projected margin of error. The total sample size was 968 participants, surpassing the minimum criteria in both data collection rounds, hence enhancing the statistical power and reliability of the study findings.

Nonetheless, as you indicated, non-response bias is a challenge in survey-based research, and we acknowledge that responders may possess distinct features compared to non-respondents, potentially affecting the generalizability of our findings. To mitigate this bias, we employed stratified random sampling to enhance representativeness and incorporated follow-up contact to diminish the probability of systematic disparities. Furthermore, we conducted a demographic analysis comparing the responder group with the university population data in Saudi Arabia, and the parallels in age and gender distribution indicate that our sample is adequately representative of the broader student population. Moreover, this point was added as a study limitation as the non-response bias remains possible.

We trust this resolves your worries and elucidates the actions implemented to mitigate non-response bias and uphold the integrity of our study.

Comment 2:

Besides, the fact that one-fourth of these respondents provided incomplete questionnaires, leading to their exclusion from the analysis, raises concerns about the credibility of the results.

Response:

Thank you for emphasizing this point. We acknowledge that the exclusion of some incomplete responses may cast doubt on the study's trustworthiness. We contend that this strategy was essential to guarantee the robustness and dependability of our findings.

The questionnaire employed in this study had 90 items aimed at thoroughly evaluating cardiovascular disease risk factors, activities, and demographic information across various dimensions of the respondents' lifestyles. Although this length probably led to a significant number of incomplete replies, we prioritized the collection of quality and reliable data over sheer numbers. Incomplete responses may have introduced inconsistencies or gaps that could undermine the validity of our results; however, by using solely complete responses, we ensured enhanced data quality and mitigated any biases arising from absent information.

To mitigate the potential stress of an extensive survey, we employed various methods to enhance students' desire to participate, including follow-up reminders. We acknowledge the constraints of partial responses and value your understanding of our choice to emphasize data completeness for thorough and dependable analysis.

Comment 3:

Additionally, highlighting that the study focuses on students is inconsistent with the broad age range of 18-50 years used in the sample. This discrepancy is especially problematic because the study investigates cardiovascular diseases, which are more prevalent in older individuals. Including older participants could dilute the study’s focus on the student population, as cardiovascular risk factors vary significantly between younger and older age groups.

Response:

Thank you for this perceptive and precise remark. We understand your concern regarding the broad age range of 18-50 years in a study focused on students.

The authors have intentionally extended the age range by including e-learning students who may differ in health status, particularly concerning non-communicable diseases, from traditional students. Cardiovascular disease risk factors related to lifestyle tend to accumulate over time, often becoming more evident with advancing age. By including a wider age range, we aimed to identify early lifestyle behaviors and risk factors in students that may have long-term consequences.

While CVD incidence increases with age, these conditions do not emerge abruptly and are influenced by the individual's lifestyle choices over time. This study sought to uncover lifestyle-related risks among younger students by observing patterns in older students as well. This approach allows for the early identification of CVD-related markers in younger students, supporting timely interventions and primary prevention efforts.
