# Supplementary material for: Risk factors for cardiovascular disease among Saudi students: Association with BMI, current smoking, level of physical activity, and dietary habits
Source: PLoS One. 2025 May 8;20(5):e0321206. doi: 10.1371/journal.pone.0321206 (PMC12061183; doi:10.1371/journal.pone.0321206)
Supplement: Attachment — Submitted filename: Authors point-by-point response.pdf [file pone.0321206.s003.pdf]

PONE-D-24-43695R1
Risk Factors for Cardiovascular Disease Among Saudi Students: Association with BMI, Current Smoking, Level of Physical Activity, and Dietary Habits
PLOS ONE

Author's point-by-point response

Reviewer #3: It is an honor to review the manuscript. This is an interesting study on “Risk Factors for Cardiovascular Disease Among Saudi Students: Association with BMI, Current Smoking, Level of Physical Activity, and Dietary Habits”.

My comments are appended.

Abstract:
In my opinion the background of the abstract needs to be more specific based on research gap. Methods need to be rewritten to add details about metrics of data and data analysis.

Response: The background and methods sections were revised, and the necessary information was incorporated.

Introduction:
In the first paragraph lots of unnecessary information. The author needs to be more specific to CVD risk and research gap in this field in Saudi Arabia. The whole introduction needs to be shortened to make it more focused.

Response: Several revisions have been made to the introduction to enhance clarity and relevance by focusing more specifically on CVD risks. Redundant information was removed, while essential details were retained to maintain the integrity of the content. Consequently, the word count was reduced from 1,147 to 841 words (after adding a new paragraph reporting the CVDs prevalence in Saudi Arabia suggested by Reviewer 2), improving brevity without compromising key information.

Methods:
The inclusion criteria are quite broad. As the study focused on CVD risk factor in students, it is better to explain the age range or student of secondary or tertiary or both were considered in the inclusion criteria.

Response: Only Saudi university students aged 18–50 years participated in this study. The research specifically targeted tertiary-level students across all academic stages, including undergraduate, postgraduate, and distance/online learning. Secondary school students were excluded from studying. The broad age range reflects the diverse demographic of Saudi university students, which includes younger traditional students and older individuals who may be pursuing postgraduate studies or returning to education later in life.
To enhance clarity, the text has been updated to explicitly state that only tertiary-level students were included and to provide a rationale for the specified age range. This explanation aligns the

inclusion criteria with the study's focus on CVD risk factors within this cohort. (Page 6; Lines 16-18)

Results:
Results have been written in the right direction.

Response: Thank you very much for your encouraging comments. Your opinion means a great deal to us and inspires us to continually surpass ourselves in striving for new heights of performance.

Discussion:
In the first paragraph the author didn’t report the key findings (specific factors found to be associated) and then needed to elaborate based on their findings.

Response: The key findings have been added at the beginning of the Discussion section (Page 19; Lines 9-18)

General comments:

The manuscript is interesting and but need improvements in the introduction and discussion section.

Response: The authors would like to thank the reviewer for their insightful and constructive comments, which significantly contributed to the enhancement of the manuscript.

Reviewer #4: An interested study investigates the CVD risk factors among Saudi using valid and suitable measures and appropriate sample size.

Abstract
Line 11 in the abstract, mention that "being between 46 and 50 years of age" but the study conducted on students. How could justify having this age group among students?

Response: Many Saudi students return to university later in life due to socioeconomic issues including profession changes or returning to school after or while working. Our study focused on 18–30-year-olds, but we also recognized non-traditional students in continuing education and professional development programs, such as those in their late 20s to early 50s. This age group was included in our research to represent the overall population and assess cardiovascular disease risk at different life stages. This is explained in several manuscript parts.

Line 15, Is oily fish consumption increased risk of CVD?

Response: No, oily fish has a protective effect, as mentioned several times throughout the manuscript. However, the insufficient consumption of oily fish by participants represents a serious concern.

The introduction is well written and comprehensively addresses topics but has some minor points that need to be addressed.

First paragraph needs more reference, and it would be better to use article not website The authors extensively using reference number 1.

Response: Modifications have been made to the introduction section to enhance clarity and increase the number of references. In the updated version, reference 1 appears only once. Furthermore, we have ensured that references are balanced and appropriately distributed. We have restricted website citations to credible and authoritative sources, including WHO, CDC, NHS, ADA, and pertinent ministries, to ensure the manuscript's quality and reliability.

Prevalence for CVD among Saudi is needed

Response: A new paragraph reporting the prevalence of CVD in Saudi Arabia have been added. (Pages 3-4; Lines 14-24 and 1-7)

Line 24, I do not think the mentioning "excessive alcohol consumption" needed when you studied Saudi population.

Response: This aspect was included in the overall discussion of behavioral risk factors for CVDs. While it may not relate directly to the Saudi population, it does provide a broader perspective for worldwide CVD risk.

Page 5 line 18, authors mentioned reference in two different styles {[24] (Aljefree and Ahmed, 2015).}. correct this

Response: Corrected.

Methods

Page 6 line 14, age from18-50 Is not age of students, either change title and remove students or remove participants above age 35.

Response: In Saudi Arabia, it is common for individuals to pursue higher education or professional studies later in life, often due to career transitions or opportunities to continue education through in-person, online, or continuing education programs. Therefore, the inclusion of participants between the ages of 35 and 50 is in line with the reality of Saudi Arabia's higher education demographics. In addition, this broader age range allows for a more comprehensive analysis of CV risk factors among participants. While we considered replacing the term "students" with "university attendees" for greater precision, this alternative could lead to confusion by including learners, academics, administrators, and staff. Therefore, we opted to retain the term "students" and provide a detailed explanation in the text to ensure clarity and consistency.

First authors mentioned the use for Saudi Food Frequency Questionnaire which include questions about demographics, smoking, physical activity, and food consumption and eating habits, but the mention that other questionnaires were used to measure demographics, smoking, physical activity,

and food consumption and eating habits with citing other reference than reference 26 for SFFQ. This quite confusing Why two questionnaires to measure the same variable Why not use the best one only? This need justification.

Response: The Saudi Food Frequency Questionnaire (SFFQ) integrates items from other validated questionnaires in certain sections. For instance, the third section of the SFFQ, which addresses physical activity and sedentary behaviors, includes items from the Arabic version of the International Physical Activity Questionnaire (IPAQ). This point has been clearly addressed in the revised version of the manuscript, specifically highlighting that the third section of the SFFQ incorporates items from the validated Arabic version of the International Physical Activity Questionnaire (IPAQ) to assess physical activity and sedentary behaviors. (Page 8; Lines 1-6)
